# Effectiveness of respiratory muscle training for patients with obstructive sleep apnea: A protocol of systematic review and meta-analysis

**DOI:** 10.1097/MD.0000000000020309

**Published:** 2020-05-15

**Authors:** Shi-Min Xue, Juan Jia, Ping Fan, Shi-Wei He

**Affiliations:** a Department of Respiratory Medicine, Yulin No. 2 Hospital, Yulin, Shaanxi 719000; b Department of Nephrology, Shaanxi Provincial Hospital of Traditional Chinese Medicine, Xi’an, Shaanxi 710003; c Department of Respiratory Medicine, Xi’an International Medical Center Hospital, Xi’an, Shaanxi 710100, China.

**Keywords:** effectiveness, obstructive sleep apnea, respiratory muscle training, safety

## Abstract

**Background:**

This study will evaluate the effectiveness and safety of respiratory muscle training (RMT) for patients with obstructive sleep apnea (OSA).

**Methods:**

Randomized controlled trials will be retrieved through electronic database searches from MEDLINE, EMBASE, Cochrane Library, CINAHL, Scopus, CBM, and CNKI from the beginning to the present. All electronic databases will be searched without any language limitation. Two researchers will independently select studies, collect data, and assess study quality, respectively. RevMan 5.3 software will be used for statistical analysis.

**Results:**

The primary outcome is severity of OSA, as measured by polysomnography or any relevant tools. The secondary outcomes are hypopnea index, apnea index, respiratory event index, respiratory disturbance index, sleep-related quality of life, and any expected or unexpected adverse events.

**Conclusion:**

The results of this study will summarize current evidence of RMT for the treatment of patients with OSA.

**Systematic review registration:**

INPLASY202040051.

## 1 Introduction

Obstructive sleep apnea (OSA) is one of the most sleep breathing disorders.^[1,2,3]^ It is characterized by hypopnea or apnea airway obstruction,^[4,5,6]^ and manifests as snoring, choking or gasping during sleep, daytime sleepiness, and startled awakening.^[7,8,9]^ It has been estimated that its prevalence in the general population varies from 6% to 38%, or to be as high as 49% among elderly population.^[10,11,12]^ Fortunately, previous studies have reported that respiratory muscle training (RMT) can be utilized for patients with OSA.^[13,14,15,16,17,18,19,20,21]^ However, no systematic review has been conducted to assess the effectiveness and safety of RMT for the treatment of OSA. Thus, this study will systematically assess the effectiveness and safety of RMT for OSA.

## 2 Methods

### 2.1 Study registration

This study was registered on INPLASY202040051, and it has been reported based on the Preferred Reporting Items for Systematic Reviews and Meta-Analysis (PRISMA) Protocol statement guidelines.^[22]^

### 2.2 Criteria for considering studies for inclusion

#### 2.2.1 Types of studies

All randomized controlled trials (RCTs) of RMT for the treatment of patients with OSA will be considered. Studies will be excluded if they are animal studies, reviews, case reports, case series, non-controlled studies, and quasi-RCTs.

#### 2.2.2 Types of participants

All patients who were diagnosed as OSA will be included. There will be no limitation in participant characteristics in this study.

#### 2.2.3 Types of interventions

In the experimental group, all patients must be treated with RMT.

In the control group, all patients can be treated by any interventions, except any forms of RMT.

#### 2.2.4 Type of outcome measurements

The primary outcome includes severity of OSA, as measured by polysomnography or any relevant tools. The secondary outcomes consist of hypopnea index, apnea index, respiratory event index, respiratory disturbance index, sleep-related quality of life, and any expected or unexpected adverse events.

### 2.3 Search strategy

#### 2.3.1 Electronic databases

This study will involve searches of MEDLINE, EMBASE, Cochrane Library, CINAHL, Scopus, CBM, and CNKI. We will include any studies published from the inception to the present. No language restriction and publication status limitation will be imposed in this study. A search strategy for Cochrane Library will be built with the support of a medical research librarian (Table 1). We will also adapt similar search strategies to the other electronic databases.

**Table 1:**
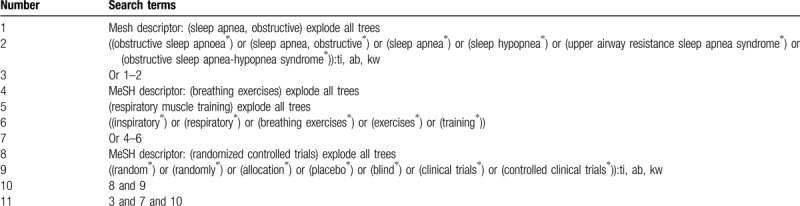
Search strategy for Cochrane Library.

#### 2.3.2 Other searches

Further studies will be obtained from reference lists of associated studies, conference abstracts, and clinical trial registries.

### 2.4 Study selection and data collection

#### 2.4.1 Study selection

Two researchers will perform selections of study independently. They will scan the titles and abstracts of all identified papers, and all irrelevant studies will be removed. For literatures where it is unclear whether the study is qualified, a further evaluation against all inclusion criteria will be checked by reading full texts of those studies. Discrepant opinions will be solved by consultation with another senior researcher. The study selection process will be presented in a flowchart.

#### 2.4.2 Data extraction

Once the RCTs for inclusion have been identified, data outlined in the pre-designed standardized data extraction sheet will be undertaken from all eligible studies. Raw data will be collected from original RCTs by two researchers: first author, publication year, location, baseline characteristics of patients, diagnostic criteria for OSA, study setting, study design, study methods, intervention details in both experimental and control group, outcomes, and safety. Any discrepancies between two researchers will be solved by another researcher through discussion to reach a consensus. We will also contact primary authors if some unclear or insufficient information occurs.

### 2.5 Risk of bias assessment

To evaluate the study quality of selected studies, we will use Cochrane Handbook for Systematic Reviews of Interventions tool to assess risk of bias assessment through seven items. The response for each one is reported as low, unclear, or high risk of bias. Two researchers will independently assess the quality of RCTs. Any divergences will be solved through discussion to make decision with the help of another researcher.

### 2.6 Statistical analysis

#### 2.6.1 Data synthesis

RevMan 5.3 software will be utilized for statistical analysis. We will pool the outcome results using risk ratio and 95% confidence intervals (CIs) for dichotomous data, and mean difference or standardized mean difference and 95% CIs for continuous data. Heterogeneity among included RCTs will be checked using *I*^2^ statistic (*I*^2^ < 50% means acceptable heterogeneity, while *I*^2^ > 50% shows high heterogeneity). We will pool the data across studies using a fixed-effects model if acceptable heterogeneity is identified, and will use a random-effect model to pool the data if there is obvious heterogeneity. Meta-analysis will be carried out if outcomes evaluated in the sufficient studies are comparable. In case of significant heterogeneity, subgroup analysis will be undertaken to check sources of obvious heterogeneity. Where appropriate, we will also present narrative synthesis in a descriptive summary.

#### 2.6.2 Reporting bias

To check reporting bias, we will perform Funnel plot and Egger regression test, if at least 10 RCTs are included.^[23]^

#### 2.6.3 Subgroup analysis

To identify possible sources of obvious heterogeneity, we will carry out subgroup analysis in accordance with the study or patient characteristics, types of interventions, controls, and outcomes.

#### 2.6.4 Sensitivity analysis

To investigate the stability of study conclusions, we will undertake sensitivity analysis by excluding low quality studies.

### 2.7 Dissemination and ethics

The results of this study will be disseminated through publication in a peer-reviewed journal. Ethical approval will not be needed for this study, because no data will be collected from individual patient.

## 3 Discussion

OSA is a very tricky disorder that interferes with a large range of adult population. Although numerous studies have reported that RMT has been utilized for the treatment of OSA, no consistent conclusions has been reached presently.^[13,14,15,16,17,18,19,20,21]^ In addition, no systematic review has performed to assess the effectiveness and safety of RMT for the treatment of OSA. Thus, this study will systematically evaluate the effectiveness and safety of RMT for OSA. The results of this study may provide encouraging evidence of RMT for the treatment of OSA.

## Author contributions

**Conceptualization:** Shi-Min Xue, Ping Fan, Shi-Wei He.

**Data curation:** Shi-Min Xue, Juan Jia, Ping Fan, Shi-Wei He.

**Formal analysis:** Juan Jia.

**Funding acquisition:** Shi-Wei He.

**Investigation:** Shi-Wei He.

**Methodology:** Shi-Min Xue, Juan Jia.

**Project administration:** Shi-Wei He.

**Resources:** Shi-Min Xue, Juan Jia, Ping Fan.

**Software:** Shi-Min Xue, Juan Jia, Ping Fan.

**Supervision:** Shi-Wei He.

**Validation:** Shi-Min Xue, Shi-Wei He.

**Visualization:** Shi-Min Xue, Juan Jia, Ping Fan, Shi-Wei He.

**Writing – original draft:** Shi-Min Xue, Shi-Wei He.

**Writing – review & editing:** Shi-Min Xue, Juan Jia, Ping Fan, Shi-Wei He.
